# Anti‐Obesity Effects of *Peucedanum Japonicum* Thunb Root Extract With Induction of Hepatic *Cyp2b10* Gene Expression in Mice

**DOI:** 10.1111/1750-3841.70524

**Published:** 2025-09-01

**Authors:** Rahmawati Aisyah, Ruj Vatanapahu, Moe Mihara, Takashi Tagawa, Mai Kanazawa, Hiroshige Kuwahara, Siyi Chen, Hidemasa Bono, Thanutchaporn Kumrungsee, Noriyuki Yanaka

**Affiliations:** ^1^ Graduate School of Integrated Sciences for Life Hiroshima University Higashi‐Hiroshima Japan; ^2^ Research Center Maruzen Pharmaceuticals, Co., Ltd Onomichi Japan

**Keywords:** cytochrome p450, fatty liver, high‐fat diet, obesity, *Peucedanum japonicum* Thunb

## Abstract

Although *Peucedanum japonicum* Thunb (PJT) has been used in traditional medicine, PJT root is considered to be agricultural waste, and its benefits have not been explored to date. This study examined the effect of PJT root extract in C57BL/6J mice fed a high‐fat diet (HFD) for 10 weeks and determined the underlying molecular mechanisms based on gene expression analyses. PJT supplementation (1% w/w) decreased body weight, white adipose tissue (WAT) mass, and hepatic triglyceride levels and downregulated inflammatory gene expression in the WAT and liver of HFD‐fed mice. Notably, dramatic increases in *Cyp2b* and *Ces2* mRNA levels, which are reportedly involved in lipid metabolism, were observed in the livers of PJT‐supplemented, HFD‐fed mice. Furthermore, short‐term PJT supplementation in HFD‐fed mice and PJT treatment in primary cultured hepatocytes suggested that PJT root extract modulates hepatic *Cyp2b* and *Ces2* mRNA expression in a direct manner. Thus, these findings demonstrate the potential of PJT root extract as a functional food for obesity and fatty liver disease.

## 1 Introduction

Obesity is defined as excessive body fat accumulation to the extent that it causes adverse effects on the body. The prevalence of obesity has been growing to epidemic proportions over the past decades owing to sedentary lifestyles, high‐fat and energy‐dense diets, and urbanization (Smyth and Heron 2006). Obesity causes various health risks, including dyslipidemia, high blood pressure, impaired insulin sensitivity, and disrupted glucose regulation. These pathological conditions further lead to long‐term complications such as cardiovascular diseases, non‐alcoholic fatty liver disease (NAFLD), and type 2 diabetes mellitus (Caprio et al. 2020). Given the increasing trend of obesity, which places a significant burden on healthcare systems, many researchers have attempted to address overweight and obesity through various strategies, including calorie‐restricted diets and anti‐obesity drugs (Tak and Lee 2021).

The use of natural sources to develop alternative anti‐obesity treatments has attracted interest from the food and pharmaceutical industries. Recent in vitro and animal studies have shown that plant phytochemicals can suppress appetite and regulate energy expenditure by inducing thermogenesis, modulating lipid metabolism, and inducing adipose tissue differentiation (Stuby et al. 2019). In animal experiments, numerous studies using a high‐fat diet (HFD) (60% calories from fat) to induce obesity and metabolic diseases in mice have revealed the anti‐obesity effects of natural bioactive compounds. For instance, natural products such as capsaicin, anthocyanin, and resveratrol have been shown to ameliorate HFD‐induced obesity by improving lipid metabolism and insulin sensitivity, reducing adipose tissue expansion and inflammation, and regulating appetite and satiety (Li et al. 2020; Esposito et al. 2015; Wang et al. 2020). These results further emphasize the potential of natural sources as alternative treatments for obesity.

The genus *Peucedanum*, belonging to the family *Apiaceae* (formerly known as *Umbelliferae*), consists of over 120 species distributed across Asia, Africa, and Europe. Plants of this genus have been used as medicines for various pathological conditions, including gastrointestinal and cardiovascular problems (Sarkhail 2014). Phytochemicals derived from different species of *Peucedanum*, including polyphenols, coumarins, and diterpenes, have traditionally gained attention for their therapeutic properties and have been studied to identify their beneficial effects. *Peucedanum japonicum* Thunb (PJT), called “chomeiso” and found in the coastal areas of Okinawa in Japan and Eastern Asia, has shown potential anti‐obesity and anti‐diabetic properties (Skalicka‐Woźniak et al. 2010; Lee et al. 2004; Hisamoto et al. 2003; Morioka et al. 2004). For instance, PJT leaves have been shown to reduce body weight gain in animal experiments (Okabe et al. 2011; Nukitrangsan et al. 2012); however, the molecular mechanisms underlying the anti‐obesity effects of PJT in vivo remain unclear. PJT roots are currently discarded as agricultural waste, and whether they exert health benefits or not has yet to be explored. In addition, different plant parts generally share a pool of bioactive compounds containing potential chemical groups, but the quantities of these compounds may vary among different parts (Chan et al. 2021; Yan et al. 2025). Thus, our study aimed to investigate the anti‐obesity effects of PJT roots in HFD‐induced mice. Our findings highlight the potential of PJT roots as alternative anti‐obesity agents and offer potential therapeutic targets for the development of functional foods related to obesity.

## 2 Materials and Methods

### 2.1 Preparation of PJT Root Extract

The roots of PJT were purchased from Yonaguni Yakusoen Co. (Okinawa, Japan). PJT root was soaked in ethanol (EtOH) and then extracted as follows. In brief, dried roots (200 g) were soaked in 1.4L of 80% EtOH and then extracted using a reflux condenser at 80°C for 1 h. After filtration, 1.4L of 80% EtOH was added to the plant residue and extracted again for 1 h. The extract solution was concentrated, freeze‐dried, and used for animal and culture cell experiments.

### 2.2 Animals and Diets

The animal experiments in this study were approved by the Animal Care and Use Committee of Hiroshima University (approval no. C‐22‐31). Mice were maintained in accordance with the Guidelines for the Care and Use of Laboratory Animals at Hiroshima University, the National Research Council's Guide for the Care and Use of Laboratory Animals, and the ARRIVE guidelines. Six‐week‐old male C57BL/J6 mice were purchased from Charles River Japan (Hino, Japan) and fed a commercial standard chow diet (MF, Oriental Yeast, Tokyo, Japan) for one week. The mice were weighed and divided into three groups: a normal diet control group (Control, *n* = 6); an HFD group (HFD, *n* = 8), in which the animals were fed a diet containing 60% calories from fat; and an HFD with PJT group (HFD/PJT, *n* = 8), in which the animals were fed an HFD supplemented with a 1% (w/w) EtOH extract of PJT root. To ensure that dietary intake in the HFD and PJT groups was the same, the animals in both groups were pair‐fed daily for 10 weeks.

To examine upregulation of hepatic gene expression levels, seven‐week‐old male CD‐1 (ICR) mice were purchased and fed a standard commercial chow diet for one week. The mice were weighed and divided into HFD (60% calories from fat) and HFD/PJT (HFD + 1% [w/w] EtOH extract of PJT root) groups. Both groups were pair‐fed for one week, and liver samples were collected for RNA extraction and gene expression analysis. All mice were housed in standard metal cages in a room with controlled temperature and humidity under a 12:12 h light/dark cycle.

### 2.3 Blood Biochemical Analysis

At the 8^th^ week of feeding, the mice were fasted for 5 h, and blood glucose levels were measured with a glucose analyzer (ACCU‐CHEK Active; Roche, Tokyo, Japan) after tail bleeding. Blood samples were collected in 1.5‐mL tubes containing 10 µL of heparin solution (MOCHIDA, Tokyo, Japan) and centrifuged at 3,000 × g for 10 min. The levels of aspartate aminotransferase (AST), alanine transaminase (ALT), serum triglycerides (TG), total cholesterol (T‐CHO), high‐density lipoprotein (HDL‐c), low‐density lipoprotein (LDL‐c), and non‐esterified fatty acids (NEFA) in the plasma on the upper layer were analyzed using a Beckman Coulter AU480 analyzer (Beckman Coulter, Krefeld, Germany) according to the manufacturer's instructions.

### 2.4 Liver Triglyceride Measurement

Lipid fractions were extracted from the liver using the Folch method. An extraction solution (chloroform:methanol, 1:2) was prepared prior to liver homogenization. Triglyceride content was determined by measuring the absorbance at 600 nm using a Multiskan FC (ThermoFisher, Tokyo, Japan) with a Triglyceride E‐Test Wako Kit (FUJIFILM, Tokyo, Japan) according to the manufacturer's instructions.

### 2.5 Primary Hepatocyte Culture

Hepatocytes were isolated from the livers of eight‐week‐old male C57BL/J6 mice by collagenase perfusion, in accordance with a previous report (Jung et al. 2020). The hepatocytes were cultured in William's medium E containing 10% fetal bovine serum (FBS), 5% penicillin–streptomycin, 0.01% bovine insulin (0.5 mg/mL), and 0.01% dexamethasone (1 mg/mL). The hepatocytes were then treated with 10 µg/mL of 1,4‐bis(3,5‐dichloro‐2‐pyridinyloxy) benzene (TCPOBOP) as a positive control for Cyp2b mRNA expression or 10 µg/mL of PJT80 root extract for 24 h. Total RNA was isolated from hepatocytes for quantitative polymerase chain reaction (qPCR) analysis.

### 2.6 DNA Microarray Analysis

Total RNA was isolated from the liver using the RNeasy Lipid Tissue Kit (Qiagen Sciences, Germantown, MD, USA) in accordance with the manufacturer's protocol. Pooled RNAs were subjected to cRNA synthesis for DNA microarray analysis with a 44 K whole mouse genome 60‐mer oligo microarray (Agilent Technologies, Palo Alto, CA, USA), as described in our previous report (Mitsumoto et al. 2017). All fluorescence labeling, hybridization, and image‐processing procedures were performed in accordance with the manufacturer's instructions. Each comparison was hybridized to two arrays employing the DyeSwap method to eliminate bias between dyes due to the difference in the efficiency of hybridization between cyanine 3‐CTP and cyanine 5‐CTP. Files and images were exported using the Agilent Feature Extraction Program (version 9.5).

### 2.7 Histological Analysis

Liver tissues were fixed in 10% phosphate‐buffered formalin and embedded in paraffin. Next, 4‐µm‐thick paraffin sections were stained with hematoxylin and eosin (H&E). The images were captured by an Olympus IX81 inverted wide microscope.

### 2.8 qPCR

Total RNA was isolated from the liver and WAT of all mice using the RNeasy Lipid Tissue Kit (Qiagen Sciences, Germantown, MD, USA). ReverTra Ace (TOYOBO, Osaka, Japan) and random hexamers (TaKaRa Bio, Kyoto, Japan) were used according to the manufacturer's instructions to synthesize cDNA. qPCR was performed on an Applied Biosystems StepOnePlus system using THUNDERBIRD Next SYBR qPCR Mix (TOYOBO, Osaka, Japan) under the previously described conditions (Mitsumoto et al. 2017). Threshold cycle (CT) values were normalized to the L19 internal reference. The primers were obtained from EuroFin Genomics (Tokyo, Japan) (Table 1).

**TABLE 1:** Primer sequences for qPCR.

Target gene	Forward (5′‐3′)	Reverse (5′‐3′)
L19	GGCATAGGGAAGAGGAAGG	GGATGTGCTCCATGAGGATGC
Adipsin	TGTACTTCGTGGCTCTGGTG	CACCTGCACAGAGTCGTCAT
Adrb3	ATGGCTCCGTGGCCTCACAGAAA	TGCGGGCGATGGCTATGATTACCAG
Atgl	TCCCACTTTAGCTCCAGGAT	AGCTTCCTCTGCATCCTCTTC
Ces2a	GCCATTATGCAGAGAGTGGAGTG	TCTGTGGCCTTGTACTGGTCG
Ces2c	TCACAACCAGATAGGTTGGCTA	GGGTGTGTACCTCAGACGATT
Ccl2	GGTCCCTGTCATGCTTCTGG	CCTTCTTGGGGGTCAGCACAG
Col1A1	CCCAAGGAAAAGAAGCACGTC	ACATTAGGGCGCAGGAAGGTCA
Cyp2b9	GGGAGTCCTGCTCATGCTCAAGT	CACCTGATCAATCTCCTTTTGGA
Cyp2b10	AAAGTCCCGTGGCAACTTCC	TCCCAGGTGCACTGTGAACA
Cyp2b13	AGCTCTCCATGACCCACAGT	GGGAGGATGGGACGTGAAGAAA
Fas	TGGGTTCTAGCCAGCAGAGAGT	ACCACCAGAGACCGTTATGC
Fgf21	ACCTGGGAGATCAGGGAGGAT	GGCCTCAGGATCAAAGTGAG
IL‐1β	TTCTTCTTTGGGTATTGCTTGG	AAGTGATATTCTCCATGAGCTT
IL6	CCTCTGGTCTTCTGGGAGTAC	AGCCACTCCTTCTGTGACTC
HSL	GGCTCACAGTTACCATCTCACC	GAGTACCTTGCTGTCCTGTCC
Leptin	GAGACCCCTGTGTCGGTTC	CTGCGTGTGTGTGAAATGTCATTG
Mmp3	TGGAGATGCTCACTTTGACG	AGAGAGCTGCACATTGGTGATG
Mpeg1	GCTTGCCTCTCTCTGCATTTCTTC	TCTTCTGCTCCAGGTTTTGGG
Pparγ	CAAGAATACCAAAGTGCGATCAA	GAGCTGGGTCTTTTCAGAATAATA
Saa1	CTCCTATTAGCTCAGTAGGTTGTG	CACTTCCAAGTTTCTGTTTATTACCC
Saa3	AAGGGTCTAGACATGTGG	ACTTCTGAACAGCCTCTCTCTG
Slc2a4	TCGTCATTGGCATTCTGGTTG	CTCCAGGTTCCGGGATGATG
SREBP‐1c	TCACAGGTCCAGCAGGTCCC	GGTACTGTGGCCAAGATGGTCC
Tnf‐α	CGTCGTAGCAAACCAGCAAG	TTGAAGAGAACCTGGGAGTAGACA

### 2.9 Statistical Analysis

All data were expressed as means ± standard error of the mean (SEM). The differences in the level of significance between the compared groups were determined using one‐way ANOVA followed by the post‐hoc Tukey test, and *p* < 0.05 was considered statistically significant (denoted appropriately in all figures).

## 3 Results and Discussion

### 3.1 PJT Root Extract Ameliorates Obesity‐Associated Phenotypes in HFD‐Fed Mice

**FIGURE 1:**
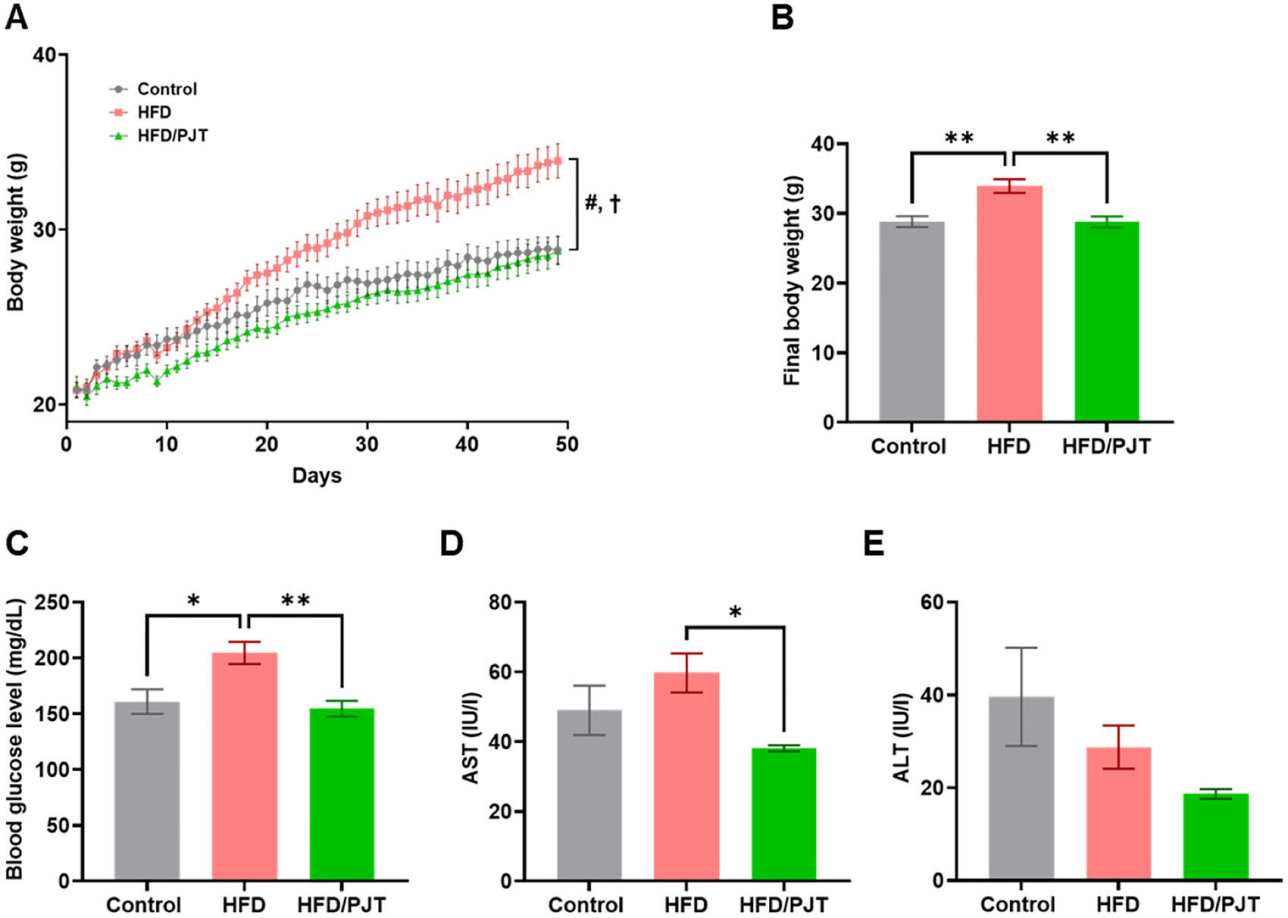
Pathological assessment of obesity in HFD‐ and dietary PJT root extract‐fed mice. (A) Change in body weight over the course of the experiment between control, HFD, and HFD/PJT mice. (B) Final body weight of control, HFD, and HFD/PJT mice. (C) Fasting blood glucose levels of control, HFD, and HFD/PJT mice. (D) Serum AST levels of control, HFD, and HFD/PJT mice. (E) Serum ALT levels of control, HFD, and HFD/PJT mice. All values are expressed as means ± SEM. Statistical analysis was performed with two‐way ANOVA (A) and one‐way ANOVA followed by the post‐hoc Tukey test (B‐E). #*p* < 0.05 (main effect HFD vs. control), †*p* < 0.05 (main effect HFD vs. HFD/PJT), *p < 0.05, **p < 0.01.

Previous studies have shown the dietary effects of PJT leaves on body weight gain and body fat accumulation in HFD mice (Nukitrangsan et al. 2012; Okabe et al. 2011); however, the in vivo molecular mechanisms underlying these effects remain unclear. Moreover, although the benefits of PJT leaf extract on obesity‐related conditions have been previously reported, the potential effects of its roots, which are considered agricultural waste, have not yet been explored. The present study examined the preventive effects of PJT root extract supplementation on HFD‐induced obesity in C57BL/6 mice and found that its supplementation significantly reduced the body weight gain in HFD‐induced obese mice (Figure 1a, b). The final body weight gain in the HFD group was 38.2 ± 2.8 g, while that in the PJT‐fed group was 32.0 ± 2.5 g (16.3% decrease vs. the HFD group; *p*  <  0.05). Consistent with the body weight results, the HFD‐induced increase in body fat mass was also significantly suppressed in the PJT‐fed group (Table 2), suggesting that the decreased body weight gain was attributed to a reduction in WAT mass. We further examined the effects of PJT root extract supplementation on serum profiles and showed lower fasting blood glucose levels in HFD/PJT mice compared to the levels in control mice (Figure 1c, Table 3). This is possibly attributed to the decreased fat mass observed in the HFD/PJT group, as studies have shown that decreased body and fat mass are associated with improved glucose homeostasis (Guilherme et al. 2008). Liver inflammatory markers (AST and ALT) were also significantly lower in the HFD/PJT group than in the HFD group (Figure 1d, e). These results suggest that PJT root extract ameliorated HFD‐induced obesity and fatty liver through metabolic regulation. As shown in Table 3, T‐CHO and LDL‐c levels in the HFD/PJT group were significantly higher than those in the HFD group. Because there is a possibility that PJT supplementation affects enterohepatic circulation of biliary acids, this study should further examine the dietary effect of PJT on bile acid metabolism by another mouse experiment with a high‐cholesterol diet.

**TABLE 2:** Effect of dietary PJT on relative organ weights in HFD‐induced obese mice.

	Units	Control	HFD	HFD/PJT
Organs				
Liver	g	1.06 ± 0.09	1.06 ± 0.11	1.06 ± 0.15
Kidneys	g	0.17 ± 0.03	0.20 ± 0.03	0.18 ± 0.02
Gastrocnemius	g	0.18 ± 0.02	0.18 ± 0.02	0.16 ± 0.01
Soleus	g	0.01 ± 0.002	0.01 ± 0.001	0.01 ± 0.001
Fat pads				
Epididymal	g	0.45 ± 0.11	2.10 ± 0.42	1.46 ± 0.44**
Perirenal	g	0.13 ± 0.05	0.76 ± 0.19	0.50 ± 0.13**
Abdominal	g	0.59 ± 0.17	2.86 ± 0.57	1.96 ± 0.56**

*Note*: Values are expressed as means ± SEM. Means in a row are statistically significantly different by one‐way ANOVA followed by the post‐hoc Tukey test.

*Abbreviations*: HFD, high‐fat diet; HFD/PJT, high‐fat diet with *Peucedanum japonicum* Thunb supplementation.

**p < 0.01 compared to the HFD group.

**TABLE 3:** Effect of dietary PJT on serum biochemicals in HFD‐induced obese mice.

	Units	Control	HFD	HFD/PJT
TG	mg/dL	19.68 ± 4.83	17.81 ± 5.08	16.64 ± 5.50
T‐CHO	mg/dL	81.74 ± 10.48	148.79 ± 34.01	187.36 ± 23.64*
HDL‐c	mg/dL	69.96 ± 9.34	116.65 ± 25.04	144.99 ± 14.53*
LDL‐c	mg/dL	9.34 ± 2.57	22.80 ± 6.47	30.73 ± 6.09*
NEFA	Eq/L	1.41 ± 0.15	1.10 ± 0.23	1.23 ± 0.14
Ketone bodies	µmol/L	313.2 ± 93.56	117.04 ± 79.52	90.57 ± 31.77

*Note*: Values are expressed as mean ± SEM. Means in a row are statistically significantly different by one‐way ANOVA followed by the post‐hoc Tukey test.

Abbreviations: HFD, high‐fat diet; HFD/PJT, high‐fat diet with Peucedanum japonicum Thunb supplementation; TG, serum triglycerides; T‐CHO, total cholesterol; HDL‐c, high‐density lipoprotein cholesterol; LDL‐c, low‐density lipoprotein cholesterol; NEFA, non‐esterified fatty acids.

*p < 0.05 compared to the HFD group.

### 3.2 PJT Root Extract Alters Adipogenesis‐ and Inflammation‐Related Gene Expression in the WAT of HFD‐Fed Mice

**FIGURE 2:**
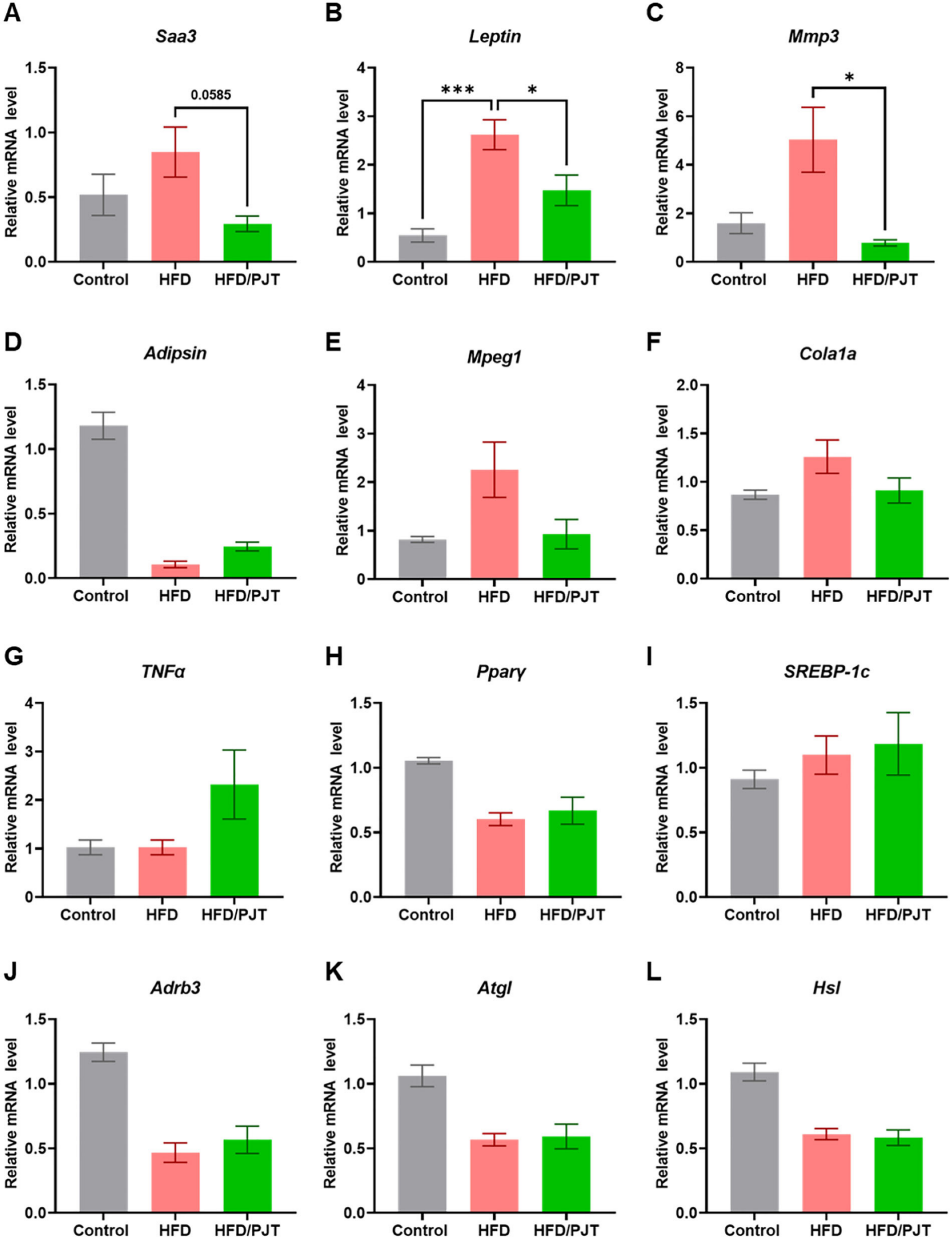
Lipid metabolism‐ and inflammatory‐related gene expression levels were altered in WAT of HFD mice supplemented with PJT root extract. qPCR results of genes related to inflammation in WAT of the HFD and HFD/PJT groups. Values are presented as means ± SEM. *p < 0.05, ***p < 0.001, as determined by the one‐way ANOVA followed by post‐hoc Tukey test.

HFD is known to increase adipocyte size and area, subsequently causing chronic inflammation with impaired secretion of various adipokines associated with immune cell infiltration, including macrophages, T lymphocytes, and B lymphocytes (Lackey and Olefsky 2016). To further understand the mechanism by which PJT root extract exerts effects on adipocytes, we examined mRNA expression levels in the WAT and showed that PJT root extract supplementation regulated the mRNA levels of genes involved in adipogenesis and inflammation (Figure 2). In particular, dietary PJT root extract supplementation decreased the mRNA levels of *Saa3* and *Mmp3*, the pro‐inflammatory markers of adipocytes, and significantly increased *Adipsin*, which plays a role in adipose tissue homeostasis and pancreatic insulin secretion under high blood glucose levels (Lo et al. 2014). These results suggest that the dietary effect of PJT root on glucose metabolism is possibly related to decreased pro‐inflammatory factor levels in WAT, emphasizing the potential anti‐inflammatory effects of PJT root supplementation on HFD‐induced adipose inflammation.

### 3.3 PJT Root Extract Upregulates the Expression of Hepatic *Cyp2b* and Carboxylesterase‐2 Genes in HFD‐Fed Mice

**FIGURE 3:**
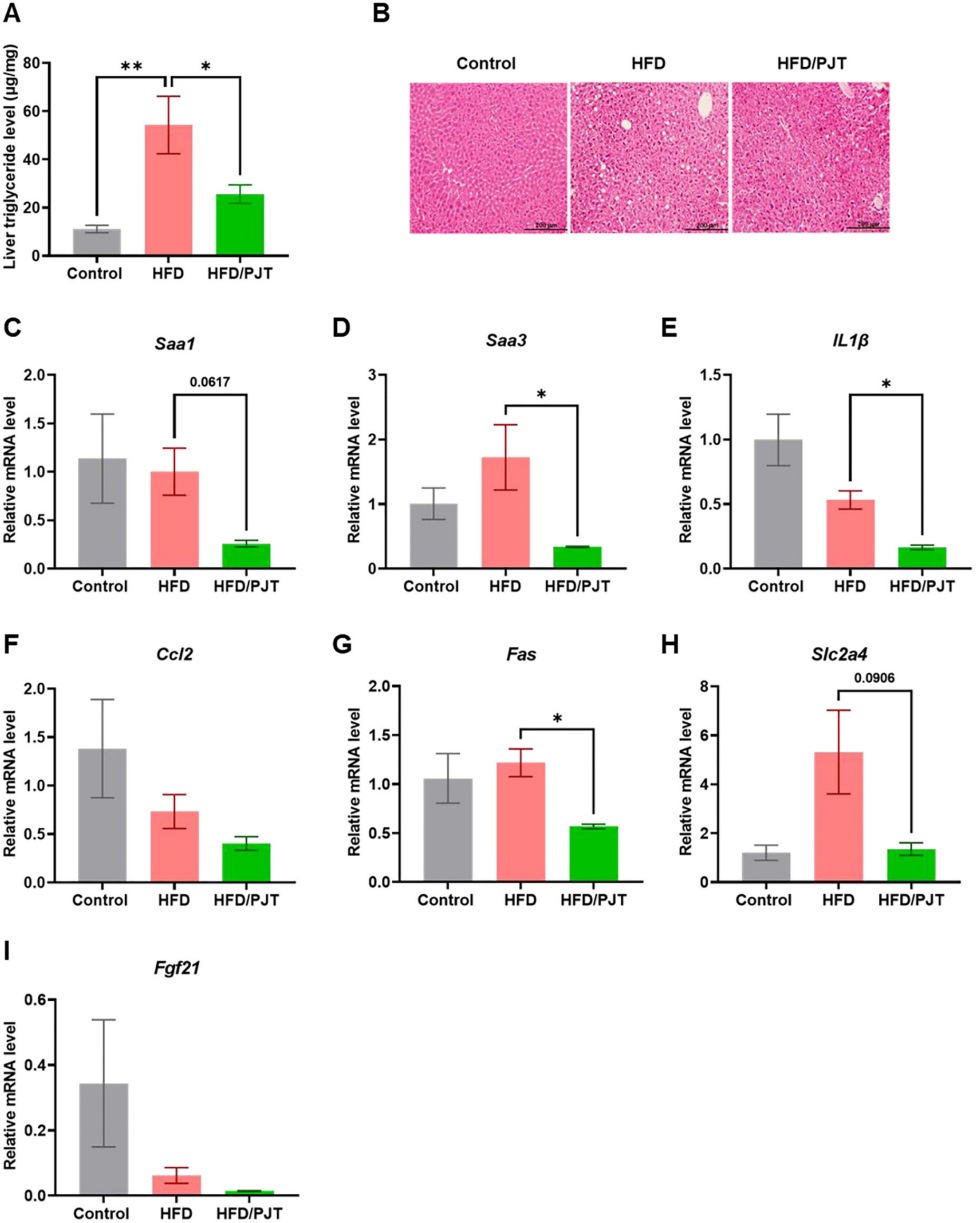
PJT root extract supplementation alleviates HFD‐induced fatty liver in obese mice. (A) Hepatic triglyceride levels in the Control, HFD, and HFD/PJT groups. (B) Representative image of H&E‐stained paraffin sections of livers. Scale bars represent 200 µm. (C‐I) Obesity‐related mRNA expression levels determined by qPCR. All values are expressed as means ± SEM. *p < 0.05, **p < 0.01, as determined by one‐way ANOVA followed by post‐hoc Tukey test.

HFD has also been shown to promote lipid accumulation in the liver, causing hepatic inflammation and NAFLD (Tsuru et al. 2020). As the HFD/PJT group showed lower liver inflammatory markers, we investigated whether dietary supplementation of PJT root extract could improve hepatic lipid metabolism in HFD‐induced obese mice. Although no difference was observed in liver weight, supplementation with PJT root extract significantly decreased liver triglyceride levels and lipid accumulation (Figure 3a, b). As impaired mRNA expression during inflammation and lipid metabolism dysfunction plays a key role in the pathogenesis of NAFLD (Hoang et al. 2019), we evaluated hepatic gene expression profiles using DNA microarray analysis and showed downregulation of the mRNA levels of fatty acid synthase (*Fas*) and inflammation‐related genes, such as serum amyloid A1 (*Saa1*) and interleukin‐1β (*Il*‐*1β*) (Figure 3c). These observations suggest that supplementation with PJT root extract has a preventive effect against liver steatosis and inflammation, indicating a possible mechanism by which dietary PJT root extract supplementation affects hepatic lipid synthesis and storage under an HFD.

Remarkably, DNA microarray data showed that dietary PJT root extract supplementation markedly increased the mRNA levels of cytochrome P450 2b (*Cyp2b*) members (*Cyp2b9*, *Cyp2b10*, and *Cyp2b13*) and carboxylesterase‐2 members (*Ces2a* and *Ces2c*). In particular, in the HFD/PJT group, the mRNA levels of *Cyp2b10* and *Ces2a* significantly increased by 42.1‐ and 2.1‐fold, respectively (Table 4). *Cyp2b* and *Ces2* participate in the metabolism of endogenous and exogenous compounds; however, both genes have recently been proposed to have novel physiological functions in lipid metabolism. Recent reports suggest that both the *Cyp2b* and *Ces2* families play preventive roles in obesity and fatty liver development by regulating triglyceride hydrolysis and fatty acid metabolism in HFD‐induced obese mice (Damiri and Baldwin 2018; Liu et al. 2021). Cytochrome P450 members were originally studied as drug‐metabolizing enzymes with numerous metabolic activities. *Cyp2b9*, *Cyp2b10*, and *Cyp2b13* are the primary hepatic *Cyp2b* genes in mice, while only *Cyp2b6* has been identified in humans (Zhao et al. 2021). Notably, recent studies have reported that *Cyp2b*‐null mice are prone to obesity when fed an HFD and show elevated hepatic triglyceride levels and altered expression of genes involved in lipid metabolism and triglyceride accumulation (Heintz et al. 2019). Moreover, the synthesis of epoxyeicosatrienoic acids from arachidonic acid, which is mediated by *Cyp2b* activity, has reportedly shown beneficial effects in preventing obesity, type 2 diabetes, and cardiovascular diseases (Waldman et al. 2016). On the other hand, *Ces2* deletion in both human primary hepatocytes and mice impairs glucose metabolism and diacylglycerol and triglyceride lipase activities, leading to liver steatosis and insulin resistance, indicating the important roles of *Ces2* gene members, including *Ces2a* and *Ces2c*, in hepatic lipid metabolism (Chalhoub et al. 2023; Ruby et al. 2017). These reports led us to a new hypothesis that dietary supplementation with PJT root extract improves obesity‐related conditions, possibly via the upregulation of *Cyp2b10* and *Ces2*, which are responsible for triglyceride hydrolytic activities.

**TABLE 4:** Selected genes differentially expressed in the livers of both the HFD and PJT groups.

Gene ID	Gene symbol	Gene description	Fold	*P*‐value
Lipid metabolism				
NM_012006	*Acot1*	acyl‐CoA thioesterase 1	1.92	0.000
NM_134246	*Acot3*	acyl‐CoA thioesterase 3, transcript variant 1	2.23	0.002
NM_134247	*Acot4*	acyl‐CoA thioesterase 4	1.49	0.049
NM_015729	*Acox1*	acyl‐Coenzyme A oxidase 1, palmitoyl, transcript variant 1	1.96	0.019
NM_007678	*Cebpa*	CCAAT/enhancer binding protein, alpha, transcript variant 1	1.38	0.004
NM_021456	*Ces1g*	carboxylesterase 1G	2.38	0.000
NM_133960	*Ces2a*	carboxylesterase 2A, transcript variant 1	2.12	0.000
NM_198171	*Ces2b*	carboxylesterase 2B	2.40	0.000
NM_145603	*Ces2c*	carboxylesterase 2C	2.64	0.000
NM_172759	*Ces2e*	carboxylesterase 2E, transcript variant 1	1.48	0.008
NM_001079865	*Ces2f*	carboxylesterase 2F, transcript variant 1	2.07	0.000
NM_009948	*Cpt1b*	carnitine palmitoyltransferase 1b, muscle	1.98	0.014
ENSMUST00000203742.1	*Crebl2*	cAMP responsive element binding protein‐like 2	1.72	0.000
NM_010000	*Cyp2b9*	cytochrome P450, family 2, subfamily b, polypeptide 9	2.27	0.000
NM_009999	*Cyp2b10*	cytochrome P450, family 2, subfamily b, polypeptide 10	42.14	0.000
NM_007813	*Cyp2b13*	cytochrome P450, family 2, subfamily b, polypeptide 13	4.65	0.000
NM_007818	*Cyp3a11*	cytochrome P450, family 3, subfamily a, polypeptide 11	4.97	0.000
NM_007820	*Cyp3a16*	cytochrome P450, family 3, subfamily a, polypeptide 16	5.42	0.000
NM_017396	*Cyp3a41a*	cytochrome P450, family 3, subfamily a, polypeptide 41a	4.58	0.000
NM_177380	*Cyp3a44*	cytochrome P450, family 3, subfamily a, polypeptide 44	4.75	0.000
NM_018887	*Cyp39a1*	cytochrome P450, family 39, subfamily a, polypeptide 1, transcript variant 1	0.67	0.000
NM_007988	*Fasn*	fatty acid synthase	0.55	0.000
NM_020013	*Fgf21*	fibroblast growth factor 21	0.06	0.000
NM_026808	*Fitm1*	fat storage‐inducing transmembrane protein 1	1.87	0.004
ENSMUST00000192506.1	*Gapdh*	glyceraldehyde‐3‐phosphate dehydrogenase	0.68	0.013
NM_018743	*Gpat4*	glycerol‐3‐phosphate acyltransferase 4	1.43	0.011
NM_013820	*Hk2*	Hexokinase 2	0.68	0.003
NM_001013770	*Lipo3*	lipase, member O3	0.59	0.021
NM_022882	*Lpin2*	lipin 2, transcript variant 2	1.83	0.007
NM_008509	*Lpl*	lipoprotein lipase	0.73	0.004
NM_025836	*Plin3*	Perilipin 3	0.66	0.024
NM_020568	*Plin4*	Perilipin 4, transcript variant 2	0.62	0.000
NM_054088	*Pnpla3*	patatin‐like phospholipase domain containing 3	0.50	0.046
NM_011480	*Srebf1*	sterol regulatory element binding transcription factor 1, transcript variant 1	0.72	0.011
Cellular signaling and communication				
NM_009696	*Apoe*	apolipoprotein E, transcript variant 1	1.65	0.048
NM_133997	*Apof*	apolipoprotein F	0.74	0.010
NM_009851	*Cd44*	CD44 antigen, transcript variant 1	0.74	0.002
X70100	*Fabp5*	mal1 mRNA for keratinocyte lipid‐binding protein.	0.39	0.036
NM_172518	*Fbxo42*	F‐box protein 42	1.44	0.002
NM_001081088	*Lrp2*	Low‐density lipoprotein receptor‐related protein 2	0.69	0.002
NM_009204	*Slc2a4*	solute carrier family 2 (facilitated glucose transporter), member 4, transcript variant 1	0.36	0.000
NM_172776	*Slc22a29*	solute carrier family 22, member 29, transcript variant 1	0.44	0.000
NM_011989	*Slc27a4*	solute carrier family 27 (fatty acid transporter), member 4	1.49	0.024
NM_013703	*Vldlr*	very low‐density lipoprotein receptor, transcript variant 1	0.56	0.000
Inflammation and immune responses				
NM_011333	*Ccl2*	chemokine (C‐C motif) ligand 2	0.51	0.000
NM_011337	*Ccl3*	chemokine (C‐C motif) ligand 3	0.56	0.000
NM_013653	*Ccl5*	chemokine (C‐C motif) ligand 5	0.59	0.007
NM_007649	*Cd48*	CD48 antigen, transcript variant 1	0.60	0.003
NM_009930	*Col3a1*	collagen, type III, alpha 1	0.73	0.028
NM_007736	*Col4a5*	collagen, type IV, alpha 5, transcript variant 2	0.58	0.026
NM_199473	*Col8a2*	collagen, type VIII, alpha 2	0.41	0.048
NM_009928	*Col15a1*	collagen, type XV, alpha 1	0.47	0.001
NM_008176	*Cxcl1*	chemokine (C‐X‐C motif) ligand 1	0.16	0.000
NM_021274	*Cxcl10*	chemokine (C‐X‐C motif) ligand 10	0.72	0.000
NM_019568	*Cxcl14*	chemokine (C‐X‐C motif) ligand 14	0.49	0.000
NM_010201	*Fgf14*	fibroblast growth factor 14, transcript variant 1	0.24	0.024
NM_019827	*Gsk3b*	glycogen synthase kinase 3 beta, transcript variant 1	1.54	0.046
NM_008361	*Il1b*	interleukin 1 beta	0.40	0.000
NM_031168	*Il6*	interleukin 6, transcript variant 1	0.33	0.001
NM_010809	*Mmp3*	matrix metallopeptidase 3	0.12	0.001
NM_001374668	*Mpeg1*	macrophage expressed gene 1, transcript variant 2	0.47	0.000
NM_009117	*Saa1*	serum amyloid A 1, transcript variant 1	0.04	0.000
ENSMUST00000210272.1	*Saa2*	serum amyloid A 2	0.06	0.000
NM_011315	*Saa3*	serum amyloid A 3	0.04	0.000
NM_011316	*Saa4*	serum amyloid A 4	0.46	0.009
NM_025429	*Serpinb1a*	serine (or cysteine) peptidase inhibitor, clade B, member 1a	1.66	0.001
NM_027971	*Serpinb12*	serine (or cysteine) peptidase inhibitor, clade B (ovalbumin), member 12, transcript variant 1	2.25	0.002

*Note*: Fold represents the average of the mRNA expression level in the livers of HFD/PJT mice relative to matched controls.

**FIGURE 4:**
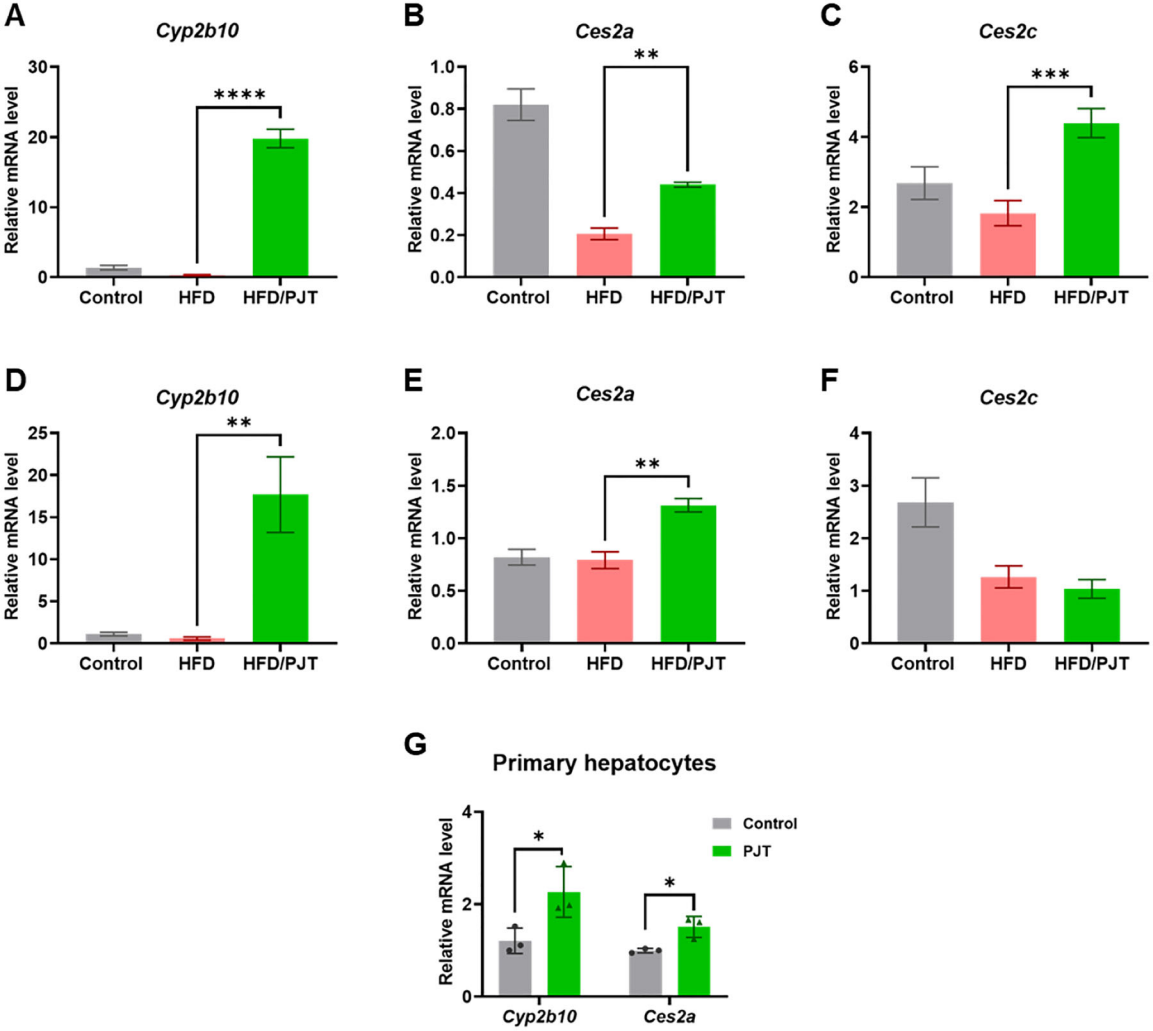
PJT root extract supplementation induces the expression of hepatic *Cyp2b* and *Ces2* genes in HFD‐fed mice. (A‐C) Gene expression of *Cyp2b10* (A), *Ces2a* (B), and *Ces2c* (C) in the control, HFD, and HFD/PJT groups under the 10‐week feeding experiment. (D‐F) Gene expression of *Cyp2b10* (D), *Ces2a* (E), and *Ces2c* (F) in the control, HFD, and HFD/PJT groups under the short‐term feeding experiment. (G) Relative mRNA expression levels of *Cyp2b10* and *Ces2a* determined by qPCR in cultured mouse primary hepatocytes. All values are expressed as means ± SEM. *p < 0.05, **p < 0.01, ***p < 0.001, and ****p < 0.0001 as determined by one‐way ANOVA followed by the post‐hoc Tukey test (A‐F) and the Student's *t*‐test (G).

Thus, we further examined whether PJT root extract supplementation directly affected hepatic *Cyp2b10* and *Ces2* mRNA expression. We found that *Cyp2b10* and *Ces2* mRNA levels increased in both 10‐week (Figure 4a‐c) and short‐term (Figure 4d‐f) treatments of PJT root extract. To determine if PJT root extract is necessary to increase *Cyp2b10* and *Ces2* mRNA levels in hepatocytes, we isolated and cultured primary hepatocytes from the livers of control mice and treated them with either TCPOBOP or 10 µg/mL PJT root extract for 24 h. TCPOBOP is a potent synthetic inducer of constitutive androstane receptor (CAR) (Baskin‐Bey et al. 2006), which is highly expressed in the liver and has been studied as a biosensor for endo‐ and xenobiotic compounds. It is also recognized as a transcription factor for *Cyp2b* genes in mice and humans (Pustylnyak et al. 2005). TCPOBOP treatment caused a 50.2‐fold increase in Cyp2b10 mRNA levels, whereas both the levels of *Cyp2b10* and *Ces2* mRNA were significantly higher in cells treated with 10 µg/mL PJT root extract than in control hepatocytes (46.59% and 33.93% increase, respectively, *p* < 0.05, Figure 4g). These suggest that PJT root extract can induce Cyp2b gene expression in a direct manner. Cyp2b expression is reportedly induced by transcription factors such as CAR, pregnane X receptor (PXR), and forkhead box protein A2 (Foxa2) (Wolfrum et al. 2003). Previous studies have highlighted CAR as the main transcription factor for *Cyp2b* gene members because CAR‐null mice showed not only decreased *Cyp2b10* mRNA expression levels but also a complete loss of *Cyp2b9* and *Cyp2b13* expression (Kumar et al. 2017; Sato et al. 2022). Furthermore, increased *Cyp2b10* mRNA levels, primarily due to CAR activation, reversed liver steatosis and improved hepatic glucose and lipid metabolism in leptin‐deficient mice (Dong et al. 2009). Interestingly, a previous report showed that nutritional factors, such as flavones, can increase *Cyp2b10* mRNA levels in HepG2 cells transfected with CAR (Yao et al. 2010). Taken together, these results suggest that the bioactive compounds in PJT root extract activate CAR to induce *Cyp2b10* gene expression. Previous studies on PJT leaf extract suggested that several coumarins, such as pteryxin and cis‐3,4‐diisovalerylkhellactone, regulate metabolic gene expression in 3T3‐L1 preadipocytes and HepG2 hepatocytes in vitro (Nugara et al. 2021; Choi et al. 2016). Therefore, further studies are needed to isolate active compounds from PJT roots to clarify the mechanisms underlying the anti‐obesity effects of PJT root extract and to propose its use as a functional food against obesity.

## 4 Conclusion

Overall, our study highlights the anti‐obesity potential of dietary PJT root extract supplementation in HFD‐fed mice by lowering triglyceride accumulation in the liver, suppressing the expansion of adipocyte area, and altering the expression of various obesity‐related genes. Dietary PJT root extract supplementation possibly exerts its effects on obesity through gene expression regulation of *Cyp2b* and *Ces2* members, which have recently been reported to be involved in the prevention of obesity and fatty liver disease in mice. In addition, our findings identified the *Cyp2b* and *Ces2* gene members as potential targets for the development of functional foods for obesity treatment.

5

**Nomenclature**
ALT: alanine aminotransferase
AST: aspartate transaminase
CAR: constitutive androstane receptor
HDL: high‐density lipoprotein
HFD: high‐fat diet
IL‐1β: interleukin 1β
NAFLD: non‐alcoholic fatty liver disease
NEFA: non‐esterified fatty acid
LDL: low‐density lipoprotein
PJT: *Peucedanum japonicum* Thunb
qPCR: quantitative polymerase chain reaction
Saa3: serum amyloid A3
T‐CHO: total cholesterol
TG: triglyceride
WAT: white adipose tissue.

## Author Contributions

**Rahmawati Aisyah**: investigation, writing–original draft, methodology, validation, writing–review and editing. **Ruj Vatanapahu**: investigation, validation, methodology. **Moe Mihara**: investigation, methodology. **Takashi Tagawa**: methodology, investigation. **Mai Kanazawa**: methodology, investigation. **Hiroshige Kuwahara**: supervision. **Siyi Chen**: methodology, investigation. **Hidemasa Bono**: methodology. **Thanutchaporn Kumrungsee**: methodology. **Noriyuki Yanaka**: conceptualization, writing–original draft, writing–review and editing, supervision.

## Conflicts of Interest

The authors declare no conflicts of interest.
